# Serotonin 6 receptor controls alzheimer’s disease and depression

**DOI:** 10.18632/oncotarget.5777

**Published:** 2015-09-22

**Authors:** Hyung-Mun Yun, Kyung-Ran Park, Eun-Cheol Kim, Sanghyeon Kim, Jin Tae Hong

**Affiliations:** ^1^ Department of Maxillofacial Tissue Regeneration, School of Dentistry and Research Center for Tooth and Periodontal Regeneration (MRC), Kyung Hee University, Seoul, Republic of Korea; ^2^ Department of Oral & Maxillofacial Regeneration, Kyung Hee University, Seoul, Republic of Korea; ^3^ College of Pharmacy and Medical Research Center, Chungbuk National University, Chungbuk, Republic of Korea; ^4^ Stanley Brain Research Laboratory, Stanley Medical Research Institute, Rockville, MD, USA

**Keywords:** 5-HT_6_R, serotonin, APBA1/2, alzheimer’s disease, depression

## Abstract

Alzheimer’s disease (AD) and depression in late life are one of the most severe health problems in the world disorders. Serotonin 6 receptor (5-HT_6_R) has caused much interest for potential roles in AD and depression. However, a causative role of perturbed 5-HT_6_R function between two diseases was poorly defined. In the present study, we found that a 5-HT_6_R antagonist, SB271036 rescued memory impairment by attenuating the generation of Aβ via the inhibition of γ-secretase activity and the inactivation of astrocytes and microglia in the AD mouse model. It was found that the reduction of serotonin level was significantly recovered by SB271036, which was mediated by an indirect regulation of serotonergic neurons via GABA. Selective serotonin reuptake inhibitor (SSRI), fluoxetine significantly improved cognitive impairment and behavioral changes. In human brain of depression patients, we then identified the potential genes, amyloid beta (A4) precursor protein-binding, family A, member 2 (APBA2), well known AD modulators by integrating datasets from neuropathology, microarray, and RNA seq. studies with correlation analysis tools. And also, it was demonstrated in mouse models and patients of AD. These data indicate functional network of 5-HT_6_R between AD and depression.

## INTRODUCTION

Alzheimer’s disease (AD) and depression are disorders of enormous and increasing public health significance [1]. Considerably higher incidences of depression are caused among aging [2]. Depression may be the initial sign of neurodegenerative disease and thus may be regarded as a risk factor for later development of AD [3]. Some studies showed higher rates of AD in those who have suffered from depression [1, 4], suggesting that the neurodegenerative process is accelerated [5–6]. Thus, depression is a potential risk factor for AD. However, the relationship between AD and depression is incompletely understood and probably complex.

Serotonin 6 receptor (5-HT_6_R) is one of the latest cloned receptors among the known 5-HT receptors and positively coupled to adenylate cyclase *via* Gs proteins [7]. The receptor has been reported to be intensively expressed in the central nervous system (CNS), especially in the striatum, hippocampus, and cortex [8]. 5-HT_6_R has shown a high affinity for antipsychotic drugs (loxapine and clozapine) as well as tricyclic antidepressant drugs (amitryptyline, clomipramine, and amoxipine) [9]. The abundant distribution in limbic region that participates in the control of mood and emotion, and the high affinity for antipsychotic and antidepressant compounds have caused much interest for the significant roles in the CNS [10–11]. In AD, considerable reductions in 5-HT_6_R density have been found in cortical areas of AD patients [12]. In preclinical studies of rodents and primates, it was reported that 5-HT_6_R antagonists enhance cognitive performance in a wide variety of learning and memory paradigms and also results in antidepressant-like activity [13–14]. In depression, preclinical data suggest potential roles for 5-HT_6_R. 5-HT_6_R antagonists have also been reported to produce antidepressant-like effects using the forced swim and tail suspension tests in both rats and mice [15–16]. Nevertheless, causative studies for functional network of 5-HT_6_R between AD and depression were not defined. We, therefore, hypothesized that 5-HT_6_R could play a critical role in link between AD and depression, which could unveil the correlation between AD and depression *via* common network of 5-HT_6_R.

In present study, we demonstrated that 5-HT_6_R regulates memory impairment and serotonin, which is reduced by GABA in the AD mouse model. We found that APBA1/2 related with amyloid beta A4 precursor protein (APP) metabolism in AD [17–19] was correlated with 5-HT_6_R in depression patients, and regulated by 5-HT_6_R in the AD mouse model, suggesting significant implications for the network of 5-HT_6_R between AD and depression.

## RESULTS

### 5-HT_6_R modulates cognitive impairment, amyloidogenesis, and neuroinflammation in the AD mouse model

**Figure 1:**
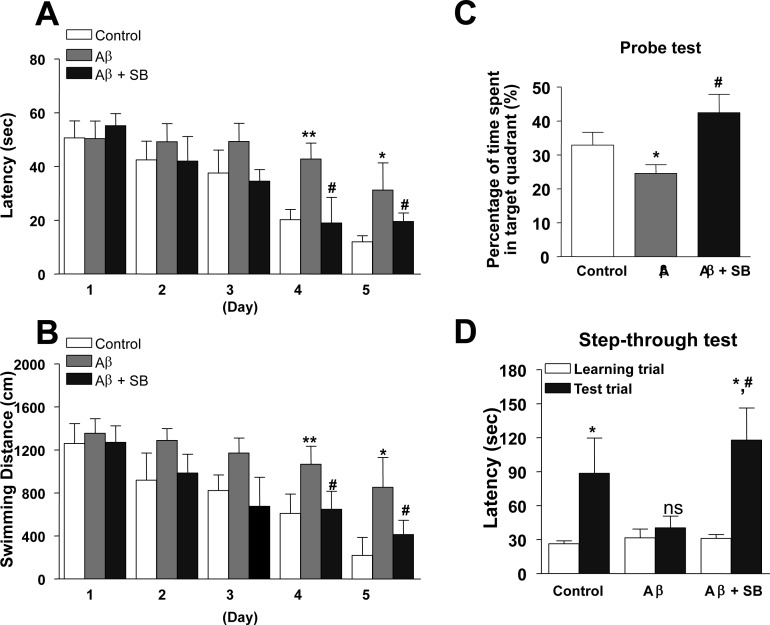
Inhibitory effects of SB271046 on memory impairment in the AD mouse model **A.–C.** Training trial was performed three times a day for 5 days. Swimming time **A.** and swimming distance **B.** to arrive at platform were automatically recorded. 24 hr after training trials, a probe test was performed. The time spent in the target quadrant and target site crossing within 60 s was represented **C.**. Each value is presented as mean ± S.E.M from 10 mice. **D.** To perform passive avoidance test, the mice were given electric shock when entered into the dark compartment for training on learning day. After one day, the retention time in illuminated compartment was recorded. Each value is presented as mean ± S.E.M. from 10 mice. *,^#^Significant difference (^*,#^*P* < 0.05 and **P < 0.01).

In order to identify whether a 5-HT_6_R antagonist, SB271046 could affect memory dysfunction in the AD mouse model, we performed behavioral tests using Morris water maze and passive avoidance tests. Mice were subjected to the Morris water maze test which is a widely known test for learning and memory. We found that Aβ_1-42_ infused mice learned more slowly than did control mice, as evidenced by slower escape latency during the acquisition training days without any difference in swim speed (Figure 1A, 1B). The learning and memory deficit was rescued after administration of SB271046 (10 mg/kg, once daily, i.p) (Figure 1A, 1B). Probe test was also confirmed the beneficial effect of SB271046 by calculating the time spent in the target quadrant zone (Figure 1C). We, next, subjected mice to the passive avoidance test that they learned to avoid a dark chamber after exposure to an electrical foot shock, followed by a retention test on the next day. Learning and memory capacities were impaired in Aβ_1-42_ infused mice, as evidenced by a significant reduction in latency to enter the dark chamber. Administration of SB271046 restored the learning and memory deficit in the passive avoidance test (Figure 1D).

**Figure 2:**
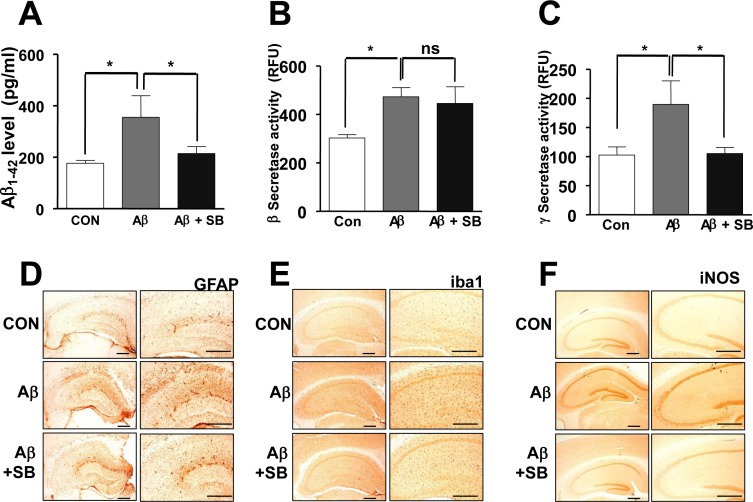
Inhibitory effects of SB271046 on Aβ1-42 generation and neuroinflammation in the AD mouse model **A.** Aβ_1-42_ level was measured in mouse brains by ELISA as described in Materials and Methods. Value is mean ± S.E.M of 8 mice. **B.**, **C.** The activities of β-secretase **B.** and γ-secretase **C.** were carried out by using each assay kit as described in Methods. Value is mean ± S.E.M of 8 mice. **D.**, **E.** The effect of SB271046 on reactive astrocytes and activated microglia cells was measured by immunohistochemical analysis. The sections of mice brain incubated with anti-glial fibrillary acidic protein (GFAP) **D.** and anti-ionized calcium binding adaptor molecule 1 (Iba1) **E.** antibodies, followed by the biotinylated secondary antibody (*n* = 8). The stained tissues were viewed with a microscope (×50 or 200). **F.** The sections of mouse brain were incubated with anti-iNOS antibody, and then followed by the biotinylated secondary antibody (*n* = 8). The resulting tissue was viewed with a microscope. ^*,#^Significant difference (^*,#^*P* < 0.05). The experiments shown in Figure 2 were repeated in triplicate with similar results.

To investigate whether SB271046 influenced amyloidogenesis, the levels of Aβ_1-42_ was determined with ELISA analysis. Elevated Aβ_1-42_ was significantly lowered by SB271046 in the AD mouse model (Figure 2A). As assessed by enzymatic cleavage assays to measure β and γ-secretase activity, the activity of β and γ-secretase significantly increased in the brain of AD mouse model (Figure 2B, 2C). The alteration in β-secretase activity was not changed by the administration of SB271046 (Figure 2B), however, -secretase activity was attenuated by SB271046 in the AD mouse model (Figure 2C). We, next, investigated inflammatory response. Consistent with a previous report [20–21], we found that GFAP-positive cells (astrocytes), Iba1-positive cells (microglia), and iNOS were elevated in the AD mouse model, while SB271046 attenuated the expression of GFAP, Iba1, and iNOS (Figure 2D-2F).

### 5-HT_6_R-mediated GABA regulation reverses cognitive impairment in the AD mouse model

**Figure 3:**
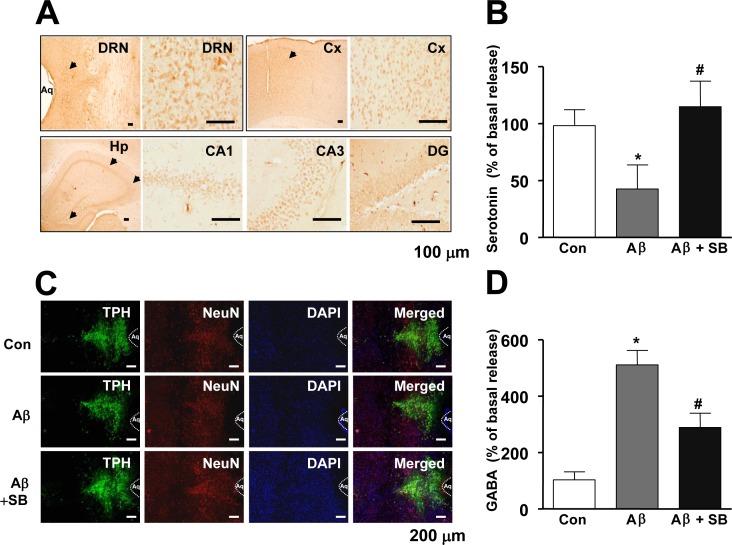
Distribution of 5-HT6R in the mouse brain, and effect of SB271046 on serotonergic neurons in the AD mouse model **A.** The DRN, Cx, and Hp (CA1, CA3, and DG) region of mice brain were analyzed using 5-HT_6_R antibodies. The stained tissues were viewed with a microscope (×50 or 200). **B.** Serotonin level was measured by ELISA as described in Materials and Methods. Value is mean ± S.E.M of 8 mice. **C.** After tissue sections were permeabilized, TPH (*green*) was immunostained with rabbit anti-TPH antibody followed by Alexa-Fluor 488-conjugated secondary antibodies, and neuronal cells (*red*) were immunostained with mouse anti-NeuN, followed by Alexa-Fluor 568-conjugated secondary antibodies. And then sections were stained with DAPI (*blue*). The *right panels* show the merged images of the *first, second*, and *third panels*. **D.** GABA level was measured by ELISA as described in Materials and Methods. Value is mean ± S.E.M of 8 mice.^ *,#^ Significant difference (^*,#^ *P* < 0.05). The experiments shown in Figure 3 were repeated in triplicate with similar results.

**Figure 4:**
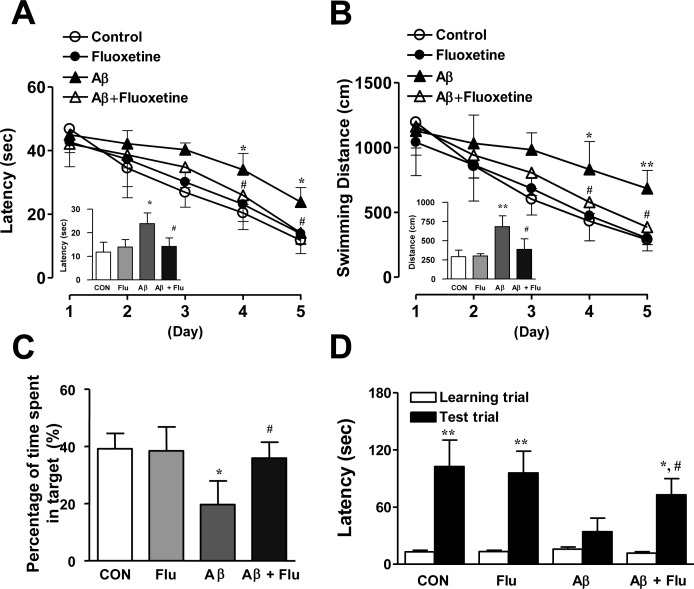
Inhibitory effects of Fluoxetine on memory impairment in Aβ1-42-infused mice model **A.–C.** Swimming time **A.** and swimming distance **B.** to arrive at platform were automatically recorded. 24 hr after training trials, a probe test was performed. The time spent in the target quadrant and target site crossing within 60 s was represented **C.**. Each value is presented as mean ± S.E.M from 10 mice. **D.** One day after the mice were given electric shock when entered into the dark compartment for training on learning day, the retention time in illuminated compartment was recorded. Each value is presented as mean ± S.E.M. from 10 mice. ^*,#^ Significant difference (^*,#^*P* < 0.05 and **P < 0.01).

We showed that 5-HT_6_R was distributed in dorsal raphe nucleus (DRN), cortex (Cx), and hippocampus (Hp) regions (CA1, CA3, DG) region in the mouse brains (Figure 3A), and it was previously reported that WAY-181187 tonically decreased neurotransmitter serotonin level in the brains, and the effect was reversed by SB271046 [22], implying that 5-HT_6_R may play important roles on serotonergic neurons. Therefore, we investigated whether 5-HT_6_R is involved in regulation of serotonergic neurons in the AD mouse model. As shown in Figure 3B, serotonin level was lowered by Aβ_1-42_ infusion, however SB271046 rescued serotonin level in AD mouse model. However, there was no difference on the cell number of TPH2-positive cells (serotonergic neurons) (Figure 3C), suggesting that 5-HT_6_R is not a direct modulator on serotonergic neurons to release serotonin. Since it has been reported that 5-HT_6_R was colocalized in GABAergic neurons [8], we measured inhibitory neurotransmitter GABA level in AD mouse model. SB271046 significantly decreased GABA level in AD mouse model (Figure 3D), suggesting 5-HT_6_R regulates serotonergic neurons and serotonin release *via* GABA. We explored whether the increased serotonin was able to improve memory impairment with same experiment of SB271046 in the AD mouse model. A selective serotonin reuptake inhibitor (SSRI), Fluoxetine showed significantly better performance on the water maze test in AD mouse model (Figure 4A, 4B). During the probe test, we calculated the time spent in the target quadrant zone during the 60 second test. The memory deficiency was significantly improved by Fluoxetine (Figure 4C). To further determine whether Fluoxetine improved the contextual memory in AD mouse model, we carried out the passive avoidance test using the step-through method. Fluoxetine significantly increased the step-through latency in AD mouse model (Figure 4D).

### APBA1/2 are correlated with 5-HT_6_R in depression patients and AD mouse model

**Figure 5:**
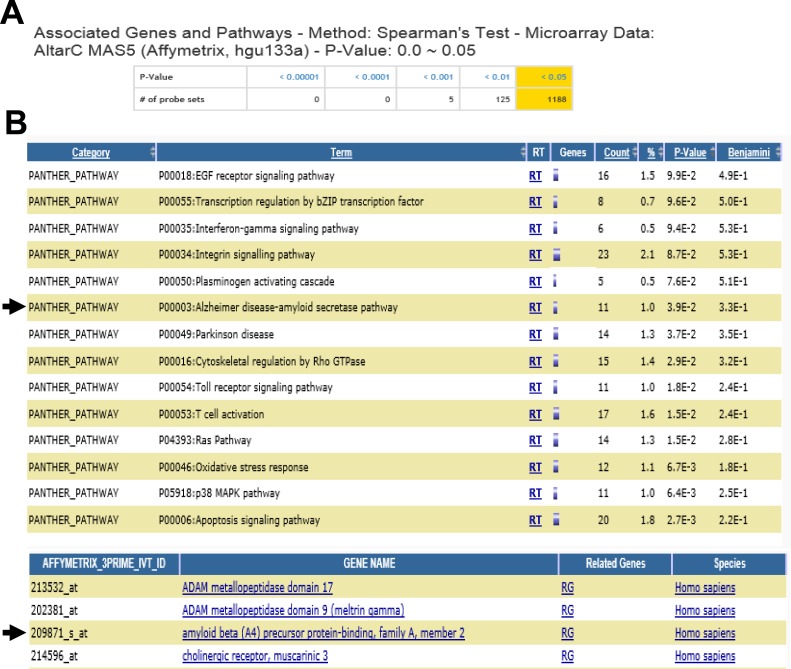
Total number of probe sets and significantly associated biological processes **A.** Screen shot captures the number of probe sets by p-values from genome-wide correlation analysis. **B.** Pathways were significantly enriched in the probe sets which were correlated with the 5-HT levels (*p* < 0.05). The functional annotation was done using an interface in the SNCID that links the SNCID (http://sncid.stanleyresearch.org) and DAVID (http://david.abcc.ncifcrf.gov/).

**Figure 6:**
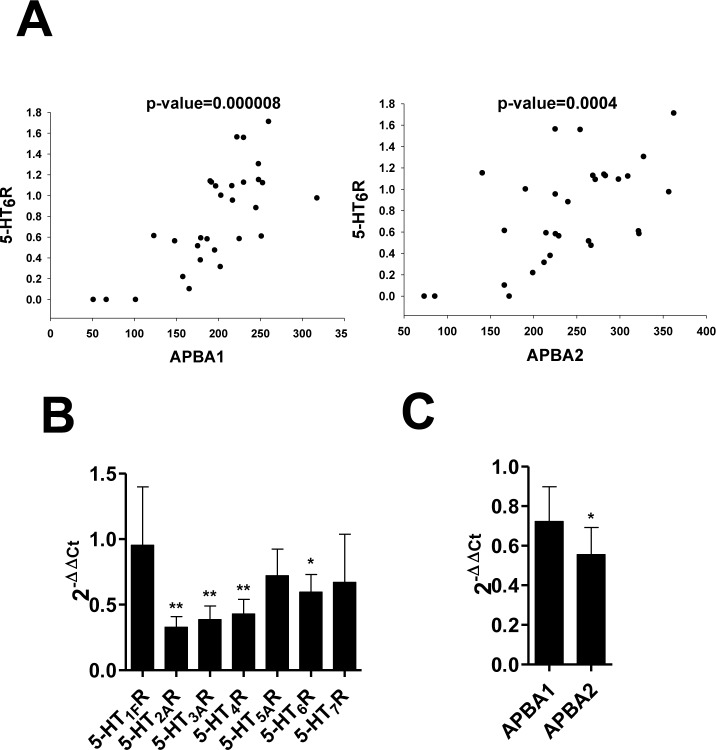
The correlation between APBA1, APBA2 and 5-HT6R in depression and AD **A** Correlation between expression levels of 5-HT_6_R and two APBA genes, APBA1 and APBA2. Gene expression levels were measured in the hippocampus of individuals with depression and unaffected controls using RNA-Seq method. (**B**., **C**.) The mRNA level of 5-HT_1_R, 5-HT_2_R, 5-HT_3_R, 5-HT_4_R, 5-HT_5_R, 5-HT_6_R, and 5-HT_7_R (**B**), and APBA1/2 (**C**) was determined by qRT-PCR in APP mutant mice of Alzheimer’s disease.

We, next, explored genes and pathways which were associated with serotonin levels in the frontal cortex of individuals with major psychiatric diseases and unaffected controls using the SNCID (sncid.stanleyresearch.org/). A total of 1188 probe sets were significantly correlated with serotonin level (Figure 5A) and a pathway related to Alzheimer disease-amyloid secretase was significantly enriched in the correlated genes (Figure 5B). Expression level of APBA2 (amyloid beta (A4) precursor protein-binding, family A, member 2) was significantly correlated with serotonin level. We further explored correlation between expression levels of two ABPA genes such as APBA1 and APBA2 and those of serotonin receptors in the hippocampus of individuals with depression and unaffected controls using RNA sequencing data which were deposited in the SNCID. Expression levels of 7 isoforms of serotonin receptor genes were analyzed, and 5-HT_6_R is closely correlated with APBA1/2 compared to other receptors (Table 1). To order to validate the correlation between 5-HT_6_R and APBA1/2, we analyzed mRNA in depression patients, AD patients, and AD mouse model. As shown in Figure 6A, 5-HT_6_R mRNA was significantly correlated with APBA1 and APBA2 in brain tissues of depression patients. In APP mutant mice of AD, it was also found that level of 5-HT_6_R mRNA was reduced in parallel with APBA2 (Figure 6B, 6C).

**Table 1:** Correlations between expression levels of isoforms of 5-HT receptors and APBA2 in the hippocampus of individuals with depression and unaffected controls

	*APBA1*	*APBA2*
*HTR1_F_*	r=0.1814	r=−0.0413
	n.s	n.s
*HTR2_A_*	r=0.3908	r=0.0785
	p=0.03	n.s
*HTR3_A_*	r=0.6551	r=0.5306
	p=0.00009	p=0.003
*HTR_4_*	r=0.7663	r=0.6061
	p=0.0000008	p=0.0004
*HTR5_A_*	r=0.6968	r=0.6175
	p=0.00002	p=0.0003
*HTR_6_*	r=0.7192	r=0.6022
	p=0.000008	p=0.0004
*HTR_7_*	r=0.7183	r=0.5303
	p=0.000008	p=0.003

Correlation analysis was performed using R. *P*-values less than 0.05 were considered significant. r, Pearson correlation coefficient; p, *P*-value; n.s, not significant.

## DISCUSSION

In the present study, we found that 5-HT_6_R improved the symptom of AD. The neurotransmitter serotonin has been known to regulate a variety of neurobehavioral processes, including cognition, memory, and mood [23–24]. Serotonergic neurotransmissions were reduced by approximately 40% in human postmortem material of AD patients [25–26]. Impaired serotonergic neurotransmission also appeared to be a central dysfunction leading to depression [1]. Disappointingly, none of the approaches have successively resolved correlation between the two diseases, although AD as a risk factor for depression has been considered and vice versa [4–5]. Actually, it is difficult to search co-effective factors related with depression in AD, since depression is complex mood disorder caused by a variety of reasons such as environmental factors, hereditary factors, and lifestyle [27–28]. Therefore, we planned a strategy to find new co-factors related with AD *via* human brain of depression patients within the category of 5-HT_6_R and serotonin. By integrating datasets from neuropathology, microarray, and RNA seq. studies with correlation analysis tools, we identified APBA1/2, known modifiers of APP processing and endocytic trafficking in AD [19]. The molecular mechanisms underlying the suppression of APP amyloidogenic metabolism by APBA1/2 have been addressed. For example, in the brains of APBA1/2 knockout mice, APP metabolism and Aβ generation were suppressed [17, 29–30]. APBA1/2 transgenic mice crossed to APPswe transgenic mice which are used as an AD mouse model demonstrated reduced Aβ deposition compared [31–32]. Recently, APBA2 was included as several known or novel modifiers in co-expression correlation network analysis of the apolipoprotein E e4 allele (APOE4, a genetic factor in AD) and AD transcriptomic changes [18]. To the best of our knowledge, it has been not reported for the involvement of APBA1/2 in depression until now. Thus, it is of interest to find the correlation of APBA1/2 with 5-HT_6_R in depression patients. We suggest that APBA1/2 is novel proteins regulated by 5-HT_6_R in AD, as well as our data imply the novel function of APBA1/2 *via* 5-HT_6_R in depression.

The dorsal raphe nucleus (DRN) is a brainstem nucleus located in the midbrain and pons, where serotonergic neurons are clustered and project a multitude of brain areas such as cortex, hippocampus, amygdala, basal ganglia, thalamus, and hypothalamus, at which they utilize many transmitters to control various physiological functions, including cognition and emotional states [33–35]. Thus, DRN has been associated with the pathophysiology of a wide spectrum of neuropsychiatric syndromes, such as depression, suicide, and AD. In the present study, it was found that 5-HT_6_R was expressed on DRN and its target region, cortex and hippocampus regions (CA1, CA3, and DG), and WAY-181187 tonically decreased serotonin level, which was attenuated by SB271046 [22]. Therefore, we expected that 5-HT_6_R affects serotonergic neurons and regulates serotonin level in the AD mouse model. However we failed to detect the significant reduction of serotonergic neuron in the AD mouse model, at which 5-HT_6_R did not affect the number of serotonergic neuron. Our results indicate that 5-HT_6_R indirectly regulates serotonin level in the AD mouse model. Previously, it was reported that 5-HT_6_R was co-localized on GABAergic neurons, implying 5-HT_6_R may modify GABAergic neurotransmission in many brain areas *via* the major inhibitory neurotransmitter GABA [8]. GABAergic afferents synapse with serotonergic neurons and have a significant effect on serotonergic neurons in the DRN [36–37]. GABA_A_ and GABA_B_ receptor agonists significantly decreased serotonin level [38–39], while GABA_A_ receptor antagonists increased serotonin level [40]. In an AD mouse model, GABA release aberrantly increased [41–42], and GABA_A_ receptor antagonists were shown to improve long term potentiation and memory [43], indicating that the aberrant GABAergic transmission exacerbates cognition in AD patients. Consistent with this observation, we found that the level of GABA was reduced by 5-HT_6_R in the AD mouse model. The observation could be validated in another aspect. Since GABAergic neurons synapse directly with cholinergic neurons [8], the regulation of 5-HT_6_R on cholinergic transmission may occur in AD. In the present study, we proved that scopolamine-induced cognitive dysfunction was attenuated by 5-HT_6_R (Supplementary Figure 1). The results support our idea for an indirect effect on serotonergic neuron *via* GABA. Thus, we suggest that 5-HT_6_R controls serotonin level *via* GABAergic neurons but not serotonergic neurons in the AD mouse model.

Amyloidogenesis posits that the aberrant metabolism and aggregation of Aβ causes a cascade of neuronal and inflammatory injury, leading to neuronal dysfunction and cell death in AD [44–46]. In the present study, we demonstrated that 5-HT_6_R attenuated the amyloid generation in the AD mouse model. The effect of 5-HT_6_R is likely attributable to the activity of γ-secretase but not β-secretase activity. We found that SB270146 and Fluoxetine reduced γ-secretase activity and did not affect β-secretase activity in the AD mouse model. Several studies support our findings. For instance, it was reported that administration of SSRIs reduced brain Aβ levels in mice [47–48], and also two components of the γ-secretase complex, presenilin (PS)-1 and nicastrin were significantly reduced in a SSRI, citalopram-treated mice [49]. Furthermore APBA1/2 directly interacted with APP and γ-secretase and impaired γ-secretase activity to inhibit Aβ metabolisms in AD [19, 50–52]. Thus, we suggest that the regulation of APBA1/2 *via* 5-HT_6_R improves cognitive dysfunction in the AD mouse model by inhibiting γ-secretase activity. Although we firstly found that the network of 5-HT_6_R-Serotonin-APBA1/2 occurs in depression patients by analyzing human depression patients, we are further studying to demonstrate the functional network of 5HT_6_R-Serotonin-APBA1/2 in a depression mouse model. In conclusion, we originally investigated the relationship of 5-HT_6_R-serotonin-APBA1/2 between AD and depression, and we suggest a potential mechanism for the network of 5HT_6_R-serotonin-APBA1/2 in the AD mouse model. Our data provide the evidence that the functional network has critical roles in both AD and depression, as well as provide the concept that the network modulation gives rise to great potential in both prevention and treatment of AD and depression.

## MATERIALS AND METHODS

### Animals

Male ICR mice (20-25 g) were purchased from Samtako, and were maintained in accordance with the National Institute of Toxicological Research of the Korea Food and Drug Administration guideline for the humane care and use of laboratory animals. All of the experimental procedures in the present study were approved by IACUC of Chungbuk National University (approval number: CBNUA-144-1001-01). Animals were housed in a room that was automatically maintained at 21~25°C and relative humidity (45~65%) with a controlled light-dark cycle. All animals had free access to food (Samyang Foods, Seoul, Korea) and water. Behavioral testing was done during the light cycle of the day (between 14.00-18.00 h). Diethyl ether was used in animal experiments as a euthanasia agent. After appropriate quantity of ether was poured onto cotton wool and allowed to evaporate and fill the chamber, the animals were placed on the ether soaked cotton wool under a mesh so that the animals do not have direct contact with the liquid chemical (ether). After euthanasia, the animal carcasses and the soaked cotton wool were removed and placed inside the chemical fume hood to allow dissipation of the chemical. The extraction system of the fume hood remained switched on for a further period of 30 minutes after the animal carcasses were handled following standard clinical waste procedures.

### AD mouse model

The anesthetized animals were placed in a sterotaxic instrument, and catheters were attached to an osmotic mini-pump (Alzet 1002, ALZA, Mountain View, CA, USA) and brain infusion kit 1 (Alzet kit 3-5 mm, ALZA, Mountain View, CA, USA) which were implanted according to the following coordinates: mouse (unilaterally): − 1.0 mm anterior/posterior, + 0.5 mm medial/lateral and − 2.5 mm dorsal/ventral. The pump contents were released over a period of 14 days consisting of 300 pmol aggregated Aβ_1-42_ (Bachem Chemical, Kashiwa St, Torrance, CA, USA) dissolved in sterile saline (0.9% NaCl) for each pump.

### Water maze test

The learning and memory capacity were assessed using two separate tests (water maze and passive avoidance tests). The water maze test is a widely accepted method for testing memory. The examination was performed using the SMART-CS (Panlab, Barcelona, Spain) program and equipment. A circular plastic pool (height 35 cm, diameter 100 cm) was filled with water (containing milk) kept at 22-25°C. An escape platform (height 14.5 cm, diameter 4.5 cm) was positioned submerged 0.5-1 cm below the surface of the water. The test was performed three times per day for 7 days. Each trial lasted for 60 s or ended as soon as the mouse reached the submerged platform and was allowed to remain on the platform for 2 s. The mice were allowed to swim until they sought the escape platform. Escape latency, escape distance, swimming speed, and swimming pattern of each mouse were monitored by a camera above the center of the pool connected to a SMART-LD program (Panlab, Barcelona, Spain). A quiet environment, consistent lighting, constant water temperature, and fixed spatial frame were maintained throughout the period of the experiment.

### Probe test

A probe test to assess memory consolidation was performed two days after the 7 days acquisition tests. In this test, the platform was removed from the tank, and the mice were allowed to swim freely. For these tests, the spent time on the target quadrant within 60 s were recorded. The time spent in the target quadrant was taken to indicate the degree of memory consolidation that had taken place after learning. The swimming pattern of each mouse was monitored by a camera above the center of the pool connected to a SMART-LD program as described above.

### Passive avoidance performance test

The passive avoidance test is also widely accepted as a simple and rapid method for testing memory. The passive avoidance response was determined using a “step-through” apparatus (Med Associates, Georgia, VT, USA) that consists of an illuminated and a dark compartment (each 20.3 × 15.9 × 21.3 cm) adjoining each other through a small gate with a grid floor of 3.175 mm stainless steel rods set 8 mm apart. One day after the probe test, the training trial was performed. The mouse was placed in the illuminated compartment facing away from the dark compartment. When the mouse moved completely into the dark compartment, it received an electric shock (1 mA, 3 s duration). Then the mouse was returned to its home cage. One day later, the mouse was placed in the illuminated compartment and the latency period to enter the dark compartment, defined as “retention,” was measured. The time when the mouse entered the dark compartment was recorded and described as step-through latency. The retention trials were set at a limit of 180 s as cut off time.

### Brain tissue collection and preservation

After behavioral tests, animals were perfused with PBS under inhaled diethyl ether anesthetization. The brains were immediately removed from skull, and the cortex and hippocampus were dissected on ice. All the brain tissues were immediately stored at −80°C, until biochemical assays were conducted.

### Western blot analysis

Cells and each area of the brain tissue were homogenized and lysed for 30 min incubation on ice. The lysates were centrifuged at 14,000 rpm for 15 min. An equal amount of total protein (20 μg) isolated from brain tissues was resolved on an sodium dodecyl sulfate (SDS) 10 or 12 % polyacrylamide gel and then transferred to a nitrocellulose membrane (Hybond ECL; Amersham Pharmacia Biotech, Piscataway, NJ, USA). Blots were blocked for 1 hr at room temperature with 5 % (w/v) non-fat dried milk in Tris-buffered saline Tween-20 [TBST: 10 mM Tris (pH 8.0) and a 150 mM NaCl solution containing 0.05 % Tween-20]. After a short wash in TBST, membranes were incubated at room temperature with specific. The blot was then incubated with the corresponding conjugated anti-mouse or anti-rabbit antibodies (1:2000, Santa Cruz Biotechnology). Immunoreactive proteins were detected with the enhanced chemiluminescence (ECL) western blotting detection system.

### Immunohistochemistry and Immunofluorescence

After being anesthetized with diethyl ether, subgroups of mice, were perfused intracardially with 50 ml saline. The brains were taken out from the skull and postfixed in 4% paraformaldehyde for 24 h at 4°C. The brains were transferred to 30 % sucrose solutions. Subsequently, brains were cut into 18 μm sections using a cryostat microtome (Leica CM1850; Leica Microsystems, Seoul, South Korea). After multiple washings in PBS, endogenous peroxidase activity was quenched by 1% hydrogen peroxide in methanol for 30 min and then cleared in PBS for 5 min. The sections were blocked for 30 min with 3% normal horse/goat serum diluted in PBS. These sections were incubated for overnight with appropriate antibodies. After washing in PBS, the sections were incubated in biotinylated goat anti-mouse/rabbit IgG antibody (1:1000 dilution, Vector Laboratories, Burlingame, CA, USA) for 1 hr at room temperature. The sections were subsequently washed and incubated with avidin-conjugated peroxidase complex (ABC kit, 1:200 dilution, Vector Laboratories, Burlingame, CA, USA) for 30 min followed by PBS washing. The peroxidase reaction was performed in PBS using 3, 3′-diaminobenzidine tetrahydrochloride (DAB, 0.02%) as the chromogen. Finally, the sections were rinsed, mounted on poly-glycine-coated slides, dehydrated, and cover-slipped for light microscopy and photography. Finally, sections were dehydrated in ethanol, cleared in xylene, and mounted with Permount (Fisher Scientific), and evaluated on a light microscopy (Olympus, Tokyo, Japan). For Immunofluorescence, sections were incubated with an anti-rabbit secondary antibody labeled with Alexa-Fluor 488 (1:400 dilution, Invitrogen) or anti-mouse secondary antibody labeled with Alexa-Fluor 568 (1:400 dilution, Invitrogen) for 2 h at room temperature. Final images were viewed on a confocal LSM 510 Laser Scanning microscope (Zeiss, Gottingen, Germany).

### Measurement of Aβ1-42

Lysates of brain tissue were obtained through protein extraction buffer containing protease inhibitor. Aβ_1-42_ level was determined using each specific enzyme-linked immunosorbent assay (ELISA) Kit (Immuno-Biological Laboratories Co., Ltd., Takasaki-Shi, Gunma, Japan). In brief, 100 μl of sample was added into the precoated plate and was incubated for overnight at 4°C. After washing each well of the precoated plate with washing buffer, 100 μl of labeled antibody solution was added and the mixture was incubated for 1 hr at 4°C in the dark. After washing, chromogen was added and the mixture was incubated for 30 min at room temperature in the dark. Finally, the resulting color was assayed at 450 nm using a microplate absorbance reader (SunriseTM, TECAN, Switzerland) after adding stop solution.

### Measurement of β- and γ-secretase activities

β-Secretase activity in all mice brains was determined using a commercially available β-secretase activity kit (R&D Systems, Minneapolis, MN, USA) and a BACE1 fluorescence resonance energy transfer assay kit (Panvera, Madison, WI, USA). Fluorescence was read at excitation and emission wavelengths of 355 and 510 nm, respectively, using a Fluostar Galaxy fluorimeter (BMG Lab Technologies, Offenburg, Germany) with Felix software (BMG Lab Technologies, Offenburg, Germany). β-Secretase activity is proportional to the fluorimetric reaction and is expressed as nanomoles per milligram protein per minute. γ-secretase activity was performed in *ex vitro* using CHAPSO-solubilized membrane fractions. Enzyme activity levels were quantified based on the description by Farmery et al [53].

### Correlation analysis using the SNCID

Correlation analyses between 5-HT levels and microarray genome-wide expression data in the frontal cortex of individuals with major psychiatric diseases and unaffected controls were conducted using the SNCID (sncid.stanleyresearch.org/) as previously described (Kim and Webster, 2010b). The SNCID is an archival and mining database which includes numerous neuropathology data, microarray data and RNA-seq data from the brain tissues from subjects in the Stanley Neuropathology Consortium. The Stanley Neuropathology Consortium contains 15 well-matched cases in each of four groups: schizophrenia, bipolar disorder, major depression, and unaffected controls. The diagnostic groups are matched for descriptive variables such as age, gender, race, post-mortem interval (PMI), mRNA quality, brain pH, and hemisphere. Demographic variables are listed in Table 2. DAVID (http://david.abcc.ncifcrf.gov/home.jsp) was used to identify the pathways that were significantly over-represented by the correlated genes. P-values less than 0.05 were considered significant.

**Table 2:** Demographic variables of samples used in the SNCID and correlation analyses

	Schizophrenia	Bipolar Disorder	Major Depression	Normal Control Subjects
Age (y)	44.2 (25-62)	42.3 (25-61)	46.5 (30-65)	48.1 (29-68)
Gender	9 M, 6 F	9 M, 6 F	9 M, 6 F	9 M, 6 F
Race	12 C, 3 A	14 C, 1 AA	15 C	14 C, 1 AA
PMI (hrs)	33.7 (12-61)	32.5 (13-62)	27.5 (7-47)	23.7 (8-42)
pH	6.1 (5.8-6.6)	6.2 (5.8-6.5)	6.2 (5.6-6.5)	6.3 (5.8-6.6)
hemisphere	6 R, 9 L	8 R, 7 L	6 R, 9 L	7 R, 8 L

M, male; F, female; C, Caucasian; AA, African American; Asian; L, left; R, right.

### Correlation analysis between 5-HT receptor genes and two APBA genes, APBA1 and APBA2 in the hippocampus using RNA-seq data

The raw RNA sequencing data (FASTAQ files, S1_Table) from the hippocampus of the Stanley Neuropathology Consortium are also available from the SNCID. The RNA-seq data from 15 depression cases and 15 unaffected controls were downloaded for use in this study. We map and quantify the RNA-seq data using the same methods as described in our previous study. In brief, all reads were mapped to the H. sapiens reference genome using TopHat v2.0.0 with the UCSC refFlat gene model annotation file (build hg18) on the -G parameter. We used the expected mean inner distance between mate paired-ends as -r parameter. TopHat calls Bowtie v0.12.7 to perform the alignment with no more than 2 mismatches. The quantification of gene expression was accomplished by HTseq v0.5.3p9 and edger package (http://www.bioconductor.org/packages/2.11/bioc/html/edgeR.html). All mapped read counts of the genes were counted by htseq-count (subprogram of HTseq) with the UCSC refFlat gene model annotation file, no strand specific option, and intersection-nonempty option. We performed Pearson correlation analysis between expression levels of 5-HT receptor genes and the two APBA genes using R.

### Statistical analysis

The data were analyzed using the GraphPad Prism version 4 program (GraphPad Software, Inc., San Diego, CA). Data are presented as mean ± S.E.M. Satistical significance was performed on the data using one-way analysis of variance (ANOVA) or unpaired Student’s *t* test. A value of *P* < 0.05 was considered to be statistically significant.

## SUPPLEMENTARY FIGURES AND TABLES


